# Liquid biopsy by NGS: differential presence of exons (DPE) in cell‐free DNA reveals different patterns in metastatic and nonmetastatic colorectal cancer

**DOI:** 10.1002/cam4.1399

**Published:** 2018-03-23

**Authors:** Susana Olmedillas‐López, Dolores C. García‐Olmo, Mariano García‐Arranz, Ramón Peiró‐Pastor, Begoña Aguado, Damián García‐Olmo

**Affiliations:** ^1^ New Therapies Laboratory Foundation Health Research Institute‐Fundación Jiménez Díaz University Hospital (FIIS‐FJD) Madrid Spain; ^2^ Experimental Research Unit General University Hospital of Albacete Albacete Spain; ^3^ Institut de Recerca Biomèdica de Lleida Centre de Recerca Experimental Biomèdica Aplicada (CREBA) Lleida Spain; ^4^ Department of Surgery School of Medicine Universidad Autónoma de Madrid (UAM) Madrid Spain; ^5^ Genomics and NGS Service Centro de Biología Molecular Severo Ochoa (CBMSO) CSIC‐UAM Madrid Spain; ^6^ Department of Surgery Fundación Jiménez Díaz University Hospital Madrid Spain

**Keywords:** Cell‐free DNA, colorectal cancer, metastasis, next‐generation sequencing, plasma

## Abstract

Next‐generation sequencing (NGS) has been proposed as a suitable tool for liquid biopsy in colorectal cancer (CRC), although most studies to date have focused almost exclusively on sequencing of panels of potential clinically actionable genes. We evaluated the clinical value of whole‐exome sequencing (WES) of cell‐free DNA (cfDNA) circulating in plasma, with the goal of identifying differential clinical profiles in patients with CRC. To this end, we applied an original concept, “differential presence of exons” (DPE). We determined differences in levels of 379 exons in plasma cfDNA and used DPE analysis to cluster and classify patients with disseminated and localized disease. The resultant bioinformatics analysis pipeline allowed us to design a predictive DPE algorithm in a small subset of patients that could not be initially classified based on the selection criteria. This DPE suggests that these nucleic acids could be actively released by both tumor and nontumor cells as a means of intercellular communication and might thus play a role in the process of malignant transformation. DPE is a new technique for the study of plasma cfDNA by WES that might have predictive and prognostic value in patients with CRC.

## Introduction

The analysis of circulating cell‐free DNA (cfDNA) is the most promising noninvasive alternative to conventional serial tissue biopsy for the analysis of the molecular features of tumors 1. Several lines of evidence support the potential clinical utility of this method of liquid biopsy in colorectal cancer (CRC), particularly at advanced stages 2. Several studies demonstrated that elevated cfDNA levels in plasma, together with a heterogeneous pattern of hotspot mutation status (including the *KRAS*,* NRAS*,* BRAF*, and *EGFR* genes, among others), provide a strong predictor of clinical prognostic value 3, 4, 5, 6. Thus, by surveillance of cfDNA in plasma, it is possible to anticipate disease progression months ahead of standard imaging follow‐up 7, 8. Similarly, cfDNA was used in a recent study to reflect tumor molecular dynamics in the drug response of metastatic patients with CRC, tracking the evolution of resistance mutations in *KRAS* pathway genes at different time points over the course of anti‐EGFR therapy 2.

Circulating tumor DNA (ctDNA) fragments represent a minor proportion of the total cfDNA and, therefore, require extremely sensitive and specific detection techniques. In this regard, next‐generation sequencing (NGS) has attracted increasing interest over the last few years. Most studies focused on NGS for liquid biopsy described targeted‐sequencing approaches, aimed at analyzing panels of genes with potential value for clinical management of different kinds of cancers, including CRC 9, 10. NGS of cfDNA in plasma has recently been applied in patients with CRC, with the goal of identifying serial changes in mutational profiles and tumor load fluctuations, facilitating early detection of recurrence 11, 12. Studies using these approaches revealed that different cancers originate detectable ctDNA alterations, the majority of which are clinically actionable by currently approved drugs. In addition, these approaches are useful for monitoring of treatment and disease progression. It must be noted, however, that tumors sometimes shed an insufficient amount of DNA for analysis, especially, but not exclusively, at early stages of disease 11, 13. Clinical sensitivity seems to be significantly affected by the surgical excision of primary tumors, mutational heterogeneity, and tumor burden, rendering analysis of mutations inefficient in cancer patients with low levels of ctDNA 14.

These observations led us to look at NGS from a different perspective. Searching for known mutations by targeted deep sequencing is the most common strategy but could be unnecessarily costly and has limited potential for tumors that shed low levels of DNA. Accordingly, we pursued an alternative approach: exome sequencing performed at a relatively shallower depth, with the goal of obtaining a more general overview of circulating DNA in plasma, including both known and unknown characteristics of cancer. The aim of this study was to compare other differential features, in terms of cfDNA rather than SNPs, in CRC patients with disseminated or localized disease, using whole‐exome sequencing. This approach represents a new strategy that broadens the scope of NGS applications in liquid biopsy, reducing costs and making it more feasible for translation to clinical scenarios. Our approach focused on identifying differential traits or genetic profiles in cfDNA, termed “differential presence of exons” (DPE), related to metastasis, that could be useful for predicting disease progress in patients with CRC.

## Materials and Methods

### Patients

Thirty patients with CRC were selected, following the criteria shown in Table 1, from January to December 2014 in the Department of General Surgery at Fundación Jiménez Díaz University Hospital, Madrid, Spain, according to a protocol approved by the Ethics Committee for Clinical Research of this Institution. Informed consent was obtained from each subject, and all investigations were performed in accordance with the principles embodied in the World Medical Association Declaration of Helsinki.

**Table 1:** Criteria for patient selection

	Non‐metastatic cohort (N)	Metastatic cohort (M)	Unclassifiable cohort (U)
Inclusion criteria	Age >18 years Candidates for elective surgery Provided informed consent Histological diagnosis: colon adenocarcinoma of enteroid pattern		
	pT1‐pT3 pN0 M0 (established by PET‐CT) R0	Any T or N M1: liver metastasis with an enteroid adenocarcinoma pattern, established histologically	pT4 and/or pN1‐pN2 M0 (established by PET‐CT)
Exclusion criteria	Previous cancers in other locations Lynch syndrome or other hereditary intestinal cancers		

R0: whole tumor was removed. PET‐CT, Positron Emission Tomography–Computed Tomography.

Subjects were classified into three groups: metastatic patients (M; *n = 10*), nonmetastatic patients (N; *n = 10*), and a group containing unclassifiable patients according to the selection criteria in Table 1 (U; *n = 10*). Briefly, we considered as unclassifiable those patients with T4 locally advanced disease and/or affected nodes, but no signs of distant metastasis affecting other organs or peritoneal carcinomatosis determined by PET‐CT (M0), except p04, who simultaneously had colorectal and bladder cancer with hepatic involvement, whose primary origin could not be determined, and p23 (T3N0M0), who had a cancer debut with intestinal obstruction requiring colonostomy. Due to the diagnostic doubts generated by these patients, they were classified in group U.

### Sample collection and processing

Blood samples were collected before surgery in EDTA tubes and centrifuged at 1800 × *g* for 10 min. Plasma obtained from the first centrifugation was centrifuged again at 3000 × *g* for 10 min, aliquoted, and stored at −80°C prior to analysis.

### Library preparation, exome capture, and sequencing

Circulating cfDNA was extracted from plasma using the QIAamp DNA Blood Mini Kit (Qiagen, Hilden, Germany). Concentration, quantity, and integrity of cfDNA were estimated prior to use. The size distribution of cfDNA fragments was determined using a 2100 BioAnalyzer (Agilent Technologies, Santa Clara, CA). Additional shearing was not performed because the majority of circulating DNA fragments in plasma are naturally short. Library preparation and specific exome capture were performed using the SeqCap EZ HGSC VCRome Kit (Roche NimbleGen, Basel, Switzerland).

Libraries were hybrid‐captured using biotinylated probes. Adapter DNA sequences were placed on both ends, yielding a total length of 126 nucleotides, and exomes were sequenced on the Illumina NextSeq500 platform (Illumina, Inc, San Diego, CA) with 75 bp paired‐end reads. Library preps and sequencing were performed at the Genomic Facility of the Scientific Park of Madrid, Spain.

### Data analysis

Around 974 M of 2 × 75 nt reads were obtained, with an average read depth of 40–80× per sample. Quality analyses were performed using the FastQC software 15. Reads were aligned against the *H. sapiens* genome (hg38) using Bowtie 16 with the following parameters: ‐v 3 ‐k 1 –best.

### Detection of differentially present exons

cfDNA sequencing data were processed as in a typical RNA‐seq pipeline, but the strategy was aimed at detecting DPE rather than gene expression. This analysis was performed using the R package “EdgeR” 17. Counts were calculated using the HTSeq‐count software 18.

Statistical methods were based on generalized linear models (glm), which are suitable for multifactor experiments of any complexity. The glm functions can test for differential expression using either likelihood ratio tests (LRT) 17 or quasi‐likelihood F‐tests (QLF) 19. The DPE for a *P*‐value of *P* ≤ 0.005 was selected. MA plots are shown for selected DPE.

### DPE clustering and principal components analysis (PCA)

To verify that all samples were behaving properly, normalized presence values were obtained for every exon for each sample using EdgeR (Counts Per Million, CPM). Once normalized presence values were calculated, they were used to cluster the samples using Ward's method 20. Principal components analysis (PCA) was performed using an R script developed in‐house, using normalized DPE presence levels.

### Random forest (RF) classification

Random forest (RF) classification was implemented with an R script using the “randomForest” package 21. Briefly, two samples from M and N were randomly selected and extracted from each group, respectively, using the eight remaining samples (16 samples in total) as a “training set” to generate a predictive algorithm. One hundred classifications were performed by iteration of this process, and the mean value of the obtained probabilities was calculated. The accuracy of the resulting model was tested by checking its ability to correctly classify previously extracted samples into their corresponding groups of origin.

### Pathway analysis

First, we used PANTHER (Protein ANalysis THrough Evolutionary Relationships, http://pantherdb.org) 22, a web‐based software for relating gene sequence to specific molecular functions, biological processes, and pathways. We submitted to PANTHER the list of differentially present genes for each group and performed a functional classification in specific biological pathways. A Wilcoxon–Mann–Whitney test was performed for paired samples using the percentage of genes classified in the same pathway against the total number of genes in each group.

On the other hand, data were analyzed with Ingenuity Pathway Analysis (IPA^®^; Qiagen, http://www.qiagenbioinformatics.com) 23, a software application for the analysis and interpretation of data derived from omics experiments. The list of over‐represented genes for each group was imported into IPA and mapped to the IPA knowledge database. We performed the core analysis for predicting pathways and molecular functions affected, based on gene expression. The significance of the association between the dataset and the specific pathways was determined by right‐tailed Fisher's exact test (*P* < 0.05). A Wilcoxon–Mann–Whitney test was performed for paired samples to analyze whether the percentage of genes from both groups were differentially distributed in the main function categories.

### Statistical analysis

Nonparametric Kolmogorov–Smirnov and Wilcoxon–Mann–Whitney tests for significance were performed in R to test differences in DNA concentration in plasma (cutoff *P*‐value of *P* < 0.05). LRT and QLF tests were performed for DPE with a *P*‐value of *P* ≤ 0.005. A Wilcoxon–Mann–Whitney test for paired samples for PANTHER analysis of pathways was performed and right‐tailed Fisher's exact test for IPA with a cutoff *P*‐value of *P* < 0.05 for both tests. A Wilcoxon–Mann–Whitney test for paired samples was also performed for gene distribution in IPA main function categories.

### Data availability

Whole‐exome sequencing data that support the findings of this study have been deposited in the European Genome‐phenome Archive (EGA) with the accession number EGAS00001002687.

## Results

### cfDNA isolation from plasma and NGS

**Figure 1:**
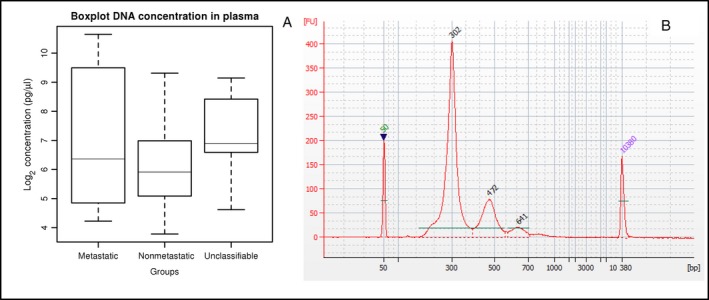
(A) Box plot of DNA concentration in plasma of patients with colorectal cancer. Median concentration of cell‐free DNA (cfDNA) in plasma was higher in metastatic patients than in nonmetastatic patients. The distribution of cfDNA concentration in unclassifiable patients shared characteristics with both classified groups. (B) Size distribution of a cfDNA library from a patient, showing a nucleosomal laddering pattern with fragment sizes of 302, 472, and 641 bp (adapter sequences included).

Circulating cfDNA was successfully extracted from all plasma samples, obtaining a variable concentration of DNA ranging from 13.76 to 1602.90 pg/*μ*L. Median DNA concentration in metastatic patients was higher than in nonmetastatic patients (Fig. 1A), although this difference was not statistically significant. Median DNA concentration in unclassifiable patients was slightly elevated with respect to the other groups. BioAnalyzer plots revealed a cfDNA size distribution with a pattern that suggested nucleosomal fragmentation. We obtained cfDNA with median fragment lengths of 173 and 342 bp, once sequencing adapter lengths (126 nt) were subtracted. One additional peak of 511 bp was observed in only two patients in our cohort (Fig. 1B).

The total number of reads per patient ranged from 45 to 87 million with a read length of 76 bp (Table S1; see supporting information for further details). Quality analyses of reads using the FastQC software (Phred+33 quality score) revealed that median and mean base quality were >28, although the quality of some bases was as low as 22. As usual, some inaccuracy was present in the first 10–11 bases.

**Figure 2:**
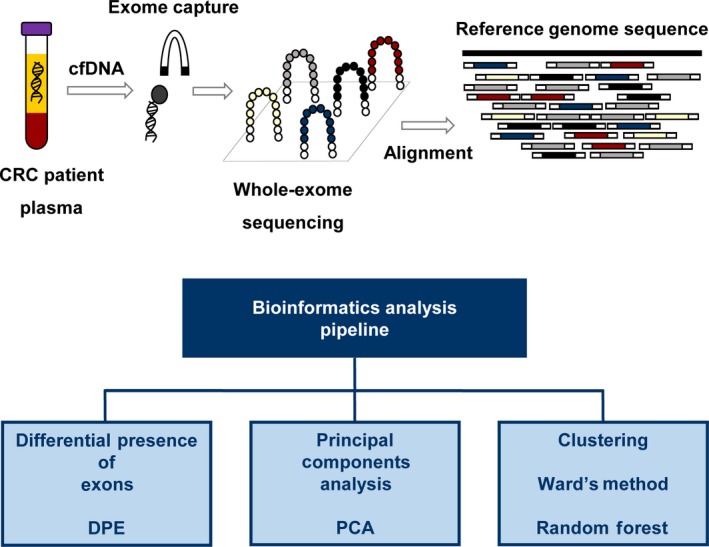
Schematic workflow of the experimental procedure. Cell‐free DNA (cfDNA) was isolated from plasma of patients with colorectal cancer (CRC). Exome capture was performed before sequencing, and the resultant reads were subsequently aligned to the reference genome sequence (hg38). A pipeline for NGS data analysis was applied to cfDNA from patients with CRC.

GC content varied between 46 and 50, and the percentage of aligned reads ranged from 64% to 78%. Thus, we considered it unnecessary to trim or filter the reads to improve quality. A schematic representation of the experimental workflow is shown in Figure 2.

### DPE analysis

We identified a set of exons whose differential presence in plasma allowed us to distinguish between groups M and N. This set of exons was later used to classify every member of the U group within group M or N.

**Figure 3:**
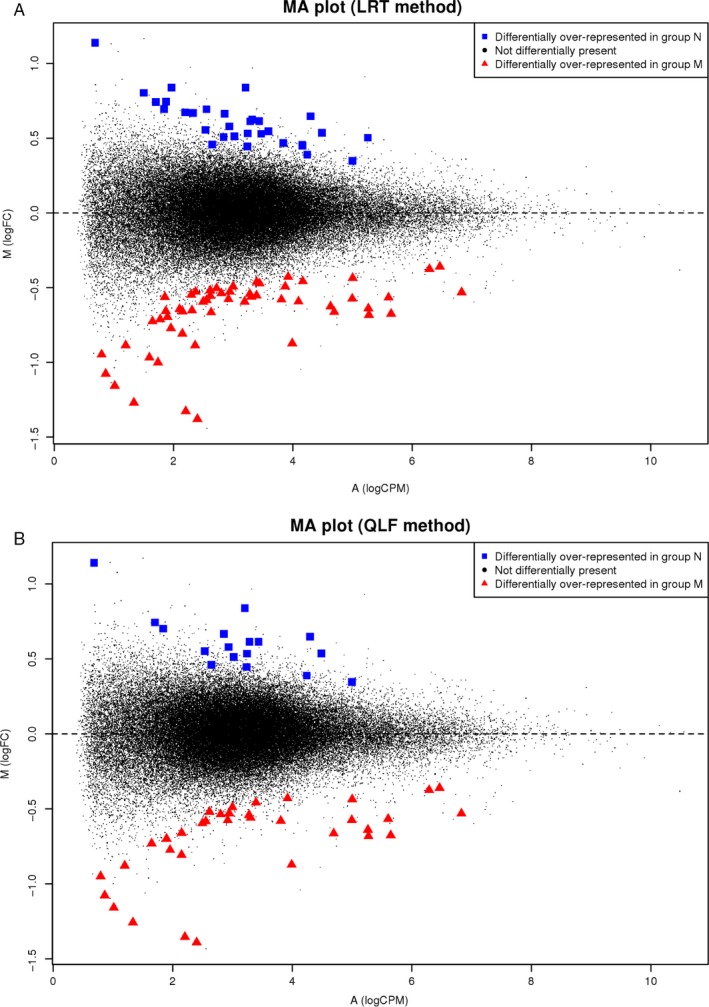
MA plots for selected differentially present exons (DPE;* P* ≤ 0.005). The log ratio of fold‐change (FC) is plotted on the *y*‐axis, and the average of the normalized counts (counts per million) is plotted on the *x*‐axis. A total of 379 exons were obtained with EdgeR, combining two different methods: (A) likelihood ratio tests (LRT) (297 exons) and (B) quasi‐likelihood f‐tests (QLF) (366 exons). Over‐represented exons in the metastatic (M) and nonmetastatic (N) groups are indicated by ▲ and ■, respectively.

Differential presence of exons was analyzed with EdgeR, using either QLF or LRT, with a threshold of *P *≤ 0.005 for M–N comparison. A total of 366 and 297 exons were obtained, respectively, yielding 379 exons overall, including unique and common exons. MA plots for selected DPE are represented in Figure 3.

A total number of 379 differentially present exons were found, 162 over‐represented in group N and 217 over‐represented in group M. Then, we examined in which genes were located these exons. Considering that some exons belong to the same gene, finally, we identified a total of 333 genes, of which 147 were over‐represented in group N and 186 in group M (Table S2; see supporting information for the complete list of genes).

### DPE clustering

**Figure 4:**
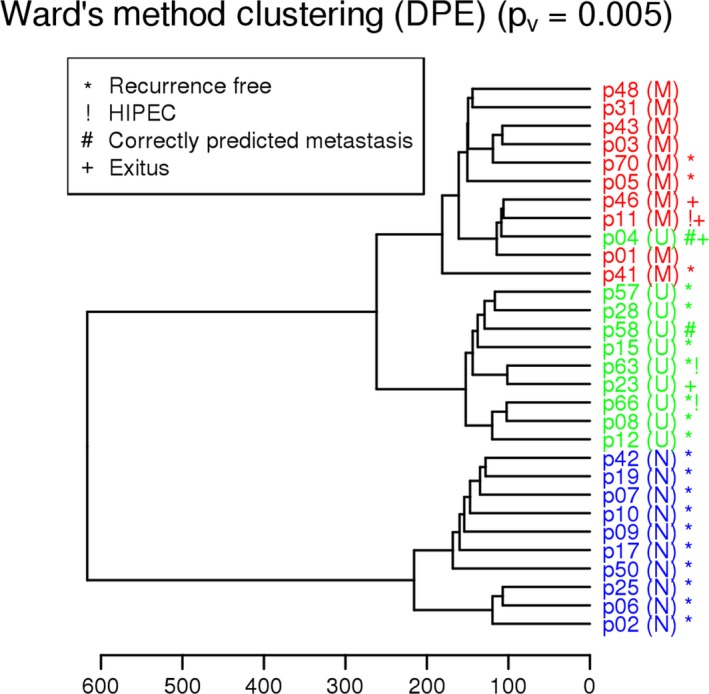
Clustering of patients by Ward's Method, using normalized values of differentially present exons (DPEs). Metastatic (M) and nonmetastatic (N) patients were clearly separated into two groups, whereas unclassifiable patients (U) were located in between, indicating that they shared traits with both groups. Patients who were recurrence‐free after the 2‐year follow‐up period are marked with an asterisk (*); remarkably, these patients tend to group together. (!) high‐risk patients treated by hyperthermic intraperitoneal chemotherapy (HIPEC); (#) correctly predicted metastasis; (+) exitus.

Clustering of normalized DPE was performed by Ward's method; the resultant tree is included in Figure 4. Patients were mostly grouped properly, maintaining the separation between the M and N samples.

**Figure 5:**
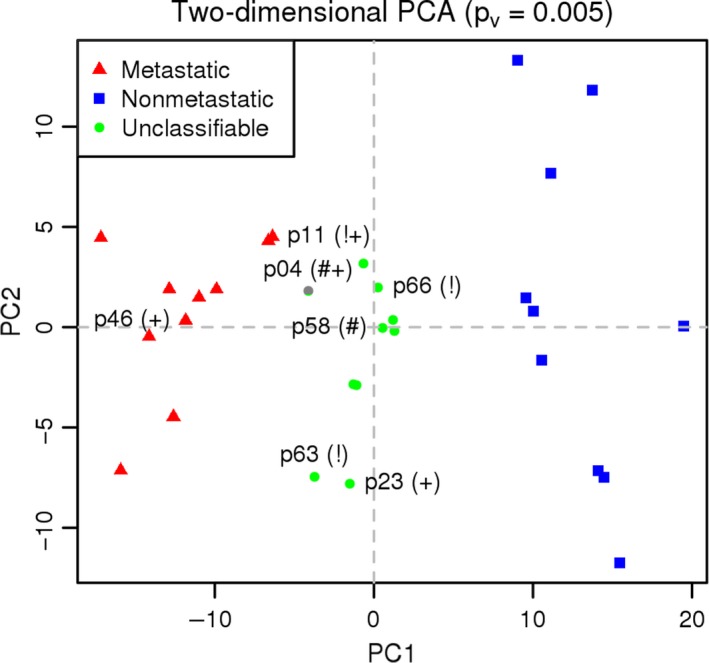
Bidimensional principal components analysis (PCA) plot. Metastatic (M) and nonmetastatic (N) patients are properly clustered and clearly separated. Unclassifiable patients (U) form another group between the limits of M and N, likely due to their intermediate characteristics. (!) high‐risk patients treated by hyperthermic intraperitoneal chemotherapy (HIPEC); (#) correctly predicted metastasis; (+) exitus.

Next, we performed PCA. Figure 5 shows a bidimensional plot with the two‐first principal components. The figure shows that groups M and N are clearly separated and clustered properly. U group patients are located between the limits of both groups, supporting the idea that patients belonging to the U group share characteristics with both metastatic and nonmetastatic patients.

These results encouraged us to develop a predictive algorithm to classify samples from patients. To achieve this goal, a RF classification was obtained after 100 iterations, extracting two randomly selected samples from each group (M and N), and generating a predictive model, with the 16 remaining samples (eight per group) as a training set. A verification test was performed to confirm that the algorithm was able to classify extracted samples into their corresponding groups of origin, by calculating the average probabilities of belonging to one group or another (Table S3; see supporting information for checking test results). The extracted samples were correctly identified, and the highest mean probability was 0.68.

Subsequently, we tried to classify the patients in group U using this algorithm, but the probabilities obtained were near 0.5 in all cases (Table S4). p04 had liver metastasis from unknown origin, and p58 was the only patient who developed metastasis during the follow‐up period. Two other patients (p63 and p66) were at very high risk of local recurrence and peritoneal metastasis according to the eligibility criteria; accordingly, they underwent prophylactic treatment (second‐look plus hyperthermic intraperitoneal chemotherapy, HIPEC). These four patients were then correctly classified by our DPE algorithm as belonging to the M group.

### Pathway analysis

We performed two different pathway analyses to gain further insight into the functional annotation of the 333 over‐represented genes. Using PANTHER, we found that most genes were not included in any specific pathway and only 139 were classified in 56 specific pathways. In addition, the distribution of genes from M and N groups in these pathways followed similar patterns (*P* = 0.3916). From the 56 identified pathways, only 17 had more than one percent of genes involved (Fig. S1).

IPA showed that over‐represented genes in both groups were related to three major biological functions: “organismal injury and abnormalities,” “cancer,” and “gastrointestinal disease.” In addition, a higher percentage of genes from group N took part in other functions different from the three major common categories mentioned above (*P* = 0.02537) (Tables S5 and S6; Fig. S2). Regarding networks, over‐represented genes in group N were associated to 11 networks whereas those in group M were related to other 14 different networks (Tables S7 and S8).

## Discussion

Historically, one of the main challenges in the analysis of circulating cfDNA is achievement of sufficient sensitivity and reproducibility despite the low concentrations of this type of DNA in plasma. The development of improved NGS methodologies probably will help to definitively overcome this limitation. In this study, we used one such methodology to analyze the plasma of CRC patients; the quality of reads and percentage of correctly aligned sequences supported the feasibility of our approach.

The size distribution reflected typical nucleosomal laddering. The predominant peak had a length around 173 bp, probably corresponding to mononucleosomal DNA, whereas other cfDNA molecules were present in multiples of this size, characteristic of a di‐ and trinucleosomal fragmentation pattern, as previously described 24. Three possible sources of cfDNA have been proposed: apoptosis, necrosis, and active release 25. However, most authors focused on cell death as the primary origin 26, 27. In this study, the oligonucleosomal laddering pattern in the size distribution and the trace quantities of sequences larger than 10,000 bp suggested that cfDNA largely originated from apoptosis, and only minimally from necrosis. These results are in agreement with previous observations 13.

Our main objective was to evaluate whole‐exome sequencing as a tool for identifying differential traits or profiles between CRC patients with disseminated (M) or localized disease (N). Interestingly, the differential features detected by our model could come from both tumor‐ and nontumor‐derived DNA. This point emphasizes the originality of our proposal and its main difference from prior studies, which focused almost exclusively on cancer‐related genetic alterations. To date, NGS has been applied to liquid biopsy for CRC by targeted deep sequencing of panels of potential clinically actionable genes carrying mutations known to be relevant to cancer development and progression, such as single‐nucleotide variants and indels, which could also be used as markers for monitoring of tumor burden 11, 12, 28, 29. Whole‐genome sequencing has also been used to search for chromosomal alterations, including copy number changes and amplifications of cancer driver genes in cfDNA of patients with CRC 13, 30, 31. For example, *MET* amplification was very recently detected by exome sequencing of the plasma of patients refractory to anti‐EGFR therapy 32.

In this study, we sought to broaden the scope of whole‐exome sequencing, using a relatively shallower depth to obtain a wider perspective on the circulating genome in plasma. This approach represents an easy, fast, noninvasive, cost‐effective, and affordable strategy for identifying patients at high risk for developing metastasis, in contrast to the personalized panels of tumor mutations previously proposed as clinical biomarkers, for which whole‐genome and ‐exome sequencing may not be cost‐effective 33.

Tracking of specific mutations by exome sequencing is hindered by several factors. One of the major hurdles is that the sensitivity of mutation detection is severely affected by the concentration of cfDNA in plasma, background noise rate, relative abundance of ctDNA, and capture efficiency 34. These approaches usually require sequencing at a high depth, which considerably increases costs, and even at a very high read depth, mutations present at extremely low levels might not be distinguishable from the sequencing background 35. Thus, the clinical utility of mutational studies based on NGS in plasma of patients with low‐shedding tumors may be limited.

Through the comparison of the M and N groups, we were able to define a set of 379 exons present at different levels in cfDNA of metastatic versus nonmetastatic patients; some of these exons were significantly over‐represented in group M, whereas others were present at higher levels in group N. This finding led to the definition of a novel concept in the field of NGS applications for liquid biopsy: “differential presence of exons” (DPE).

Differential detection of exons suggests differential release of cfDNA, supporting the idea of an active release of nucleic acids by cells, perhaps as a means of intercellular communication. Horizontal transfer of DNA between cells has been proposed as a pivotal mechanism in the development of metastasis both *in vitro* and *in vivo*, a phenomenon called genometastasis 36, 37, 38, 39. Consistent with this, several publications support the idea that various cell types selectively release newly synthesized DNA, probably associated with lipid and protein complexes, as an aspect of homeostatic processes 40, 41. In some cases, these nucleoprotein complexes also exhibit transforming activity 42. Thus, the cfDNA found in plasma of our patients could have been, to a greater or lesser extent, actively secreted by both tumor and nontumor cells and may contribute to metastasis.

In a recent study, read depth coverage patterns associated to nucleosome occupancy at promoters allowed for the identification of expressed and silent genes. Using machine learning, expression signatures were inferred, and cancer driver genes of metastatic patients were classified by copy number gains 43. Whether our differential presence profiles are in some way related to the nucleosomal fragmentation pattern and changes in gene expression or silencing should be further investigated.

When pathway analyses were performed, we found that genes from both groups (N and M) were distributed in the same pathways. Of note, IPA analysis showed that genes from both groups are affecting three major categories of functions, but these genes are involved in different networks.

For the purposes of this project, we assumed that the terms “differentially expressed” and “differentially present” were equivalent, although the experiment did not involve RNA‐seq. To the best of our knowledge, this is the first study to apply this kind of approach to analysis of cfDNA from patients with cancer. The resulting profiles of DPE were used to cluster and classify M and N patients, and this information was further processed to develop a DPE algorithm that is capable of providing a predictive model. Thus, in our series, M and N were correctly clustered and clearly separated, whereas unclassifiable patients were intermediate between the two other groups. Interestingly, the only two metastatic patients from the U group as well as two other patients at high risk subjected to second‐look HIPEC, a radical prophylactic treatment aimed at preventing recurrence and progression, were correctly classified by the DPE algorithm, supporting the potential predictive value of this model. Notably, the probabilities of belonging to one group or another were always near 0.5, suggesting that patients of group U shared common characteristics of both groups M and N. In fact, in the verification test, the probabilities obtained for M and N samples randomly selected from the training set and correctly classified by the algorithm were at most 0.68.

These results encourage us to design further studies to confirm the predictive and prognostic value of our model, as well as to evaluate its utility for early identification of high‐risk patients.

## Conflict of Interest

A patent application related to the “differential presence of exons” has been submitted to the European Patent Office (EPO). This patent is entitled “methods for identifying cancer patients at high risk of developing metastasis” (EP17382659.5). The authors have no other conflict of interests to declare.

## Supporting information


**Figure S1.** Complete list of 56 specific pathways from PANTHER public database (identified by PANTHER specific codes) in which 139 genes (to which differentially present exons belong both from groups M and N) are classified.
**Figure S2.** Complete list of main function categories from IPA.**Table S1.** cfDNA isolation from plasma and NGS.
**Table S2.** Complete list of over‐represented genes for groups N (non‐metastatic) and M (metastatic).
**Table S3.** Verification test.
**Table S4.** Classification of unclassifiable (U) patients by the algorithm.
**Table S5.** Complete list of main IPA function categories affected by over‐represented genes in group M with their associated range of *P*‐values.
**Table S6.** Complete list of main IPA function categories affected by over‐represented genes in group N with their associated range of *P*‐values.
**Table S7.** Complete list of IPA networks in which over‐represented genes in group M are involved with their associated scores (based on the number of over‐represented genes in the network with respect to the global size of that network).
**Table S8.** Complete list of IPA networks in which over‐represented genes in group N are involved with their associated scores (based on the number of over‐represented genes in the network with respect to the global size of that network).
