# Supplementary material for: Liquid biopsy by NGS: differential presence of exons (DPE) in cell‐free DNA reveals different patterns in metastatic and nonmetastatic colorectal cancer
Source: Cancer Med. 2018 Mar 23;7(5):1706–16. doi: 10.1002/cam4.1399 (PMC5943476; doi:10.1002/cam4.1399)

**Supporting Information**

**Supporting Table 1:** cfDNA isolation from plasma and NGS. Total number of reads, GC content, DNA concentration in plasma and cell-free DNA size distribution (fragment lengths in base pairs) for each individual patient is shown.

| **Patient ID** | **Reads** | **G+C** | **DNA (pg/µL)** | **Peak 1 (bp)** | **Peak 2 (bp)** | **Peak 3 (bp)** | **Group** |
| --- | --- | --- | --- | --- | --- | --- | --- |
| p01 | 74143114 | 49 | 73.97 | 298 | 459 |  | M |
| p02 | 86815250 | 50 | 635.47 | 293 |  |  | N |
| p03 | 55673798 | 48 | 18.71 | 297 | 460 |  | M |
| p04 | 87651134 | 48 | 566.00 | 302 |  |  | U |
| p05 | 50063772 | 48/47 | 28.84 | 295 | 464 |  | M |
| p06 | 60477358 | 49 | 100.27 | 295 | 462 |  | N |
| p07 | 54333354 | 48/47 | 73.55 | 298 | 469 |  | N |
| p08 | 77724428 | 48 | 151.34 | 296 | 464 |  | U |
| p09 | 50174462 | 47/46 | 33.99 | 297 | 475 |  | N |
| p10 | 56406050 | 47/46 | 23.99 | 297 | 469 |  | N |
| p11 | 55199556 | 49 | 1602.9 | 295 |  |  | M |
| p12 | 47482676 | 48 | 129.36 | 297 | 467 |  | U |
| p15 | 54307914 | 47 | 96.29 | 296 | 467 |  | U |
| p17 | 73159248 | 47 | 49.36 | 299 | 466 |  | N |
| p19 | 84446166 | 48 | 126.72 | 299 | 472 |  | N |
| p23 | 73253014 | 46 | 443.75 | 299 | 471 | 632 | U |
| p25 | 75216530 | 50 | 250.77 | 296 | 463 |  | N |
| p28 | 73314574 | 47 | 100.05 | 300 | 474 |  | U |
| p31 | 45252722 | 46 | 1128.07 | 298 |  |  | M |
| p41 | 46336662 | 47 | 23.81 | 299 | 478 |  | M |
| p42 | 65112448 | 48 | 45.97 | 297 | 466 |  | N |
| p43 | 72144422 | 48/49 | 122.37 | 303 | 478 |  | M |
| p46 | 60040810 | 48 | 721.52 | 304 | 453 |  | M |
| p48 | 83631372 | 49 | 91.48 | 310 | 466 |  | M |
| p50 | 53433962 | 46 | 13.76 | 307 | 481 |  | N |
| p57 | 69015600 | 48 | 80.94 | 301 | 469 |  | U |
| p58 | 63934496 | 48 | 24.57 | 304 | 474 |  | U |
| p63 | 52804622 | 46 | 342.28 | 302 | 472 | 641 | U |
| p66 | 78342024 | 48 | 108.48 | 304 | 481 |  | U |
| p70 | 68775536 | 49/48 | 63.89 | 299 | 466 |  | M |
| Group M = |  |  |  |  |  |  | 10 |
| Group N = |  |  |  |  |  |  | 10 |
| Group U = |  |  |  |  |  |  | 10 |
| Median |  |  | 98.17 | 299 | 468 | 637 |  |

**Supporting Table 2:** Complete list of over-represented genes for groups N (non-metastatic) and M (metastatic). Information about the gene ID, entrez gene name and symbol is provided. Flags: D, duplicated genes.

| **Group** | **ID** | **Flags** | **Symbol** | **Entrez Gene Name** |
| --- | --- | --- | --- | --- |
| **Over-represented in group N** | ENSG00000144820 |  | ADGRG7 | adhesion G protein-coupled receptor G7 |
|  | ENSG00000072364 |  | AFF4 | AF4/FMR2 family member 4 |
|  | ENSG00000168710 |  | AHCYL1 | adenosylhomocysteinase like 1 |
|  | ENSG00000238278 |  | ALG1L6P | asparagine-linked glycosylation 1-like 6, pseudogene |
|  | ENSG00000077420 |  | APBB1IP | amyloid beta precursor protein binding family B member 1 interacting protein |
|  | ENSG00000186466 |  | AQP7P1 | aquaporin 7 pseudogene 1 |
|  | ENSG00000104043 |  | ATP8B4 | ATPase phospholipid transporting 8B4 (putative) |
|  | ENSG00000182272 |  | B4GALNT4 | beta-1,4-N-acetyl-galactosaminyltransferase 4 |
|  | ENSG00000186716 |  | BCR | BCR, RhoGEF and GTPase activating protein |
|  | ENSG00000157388 |  | CACNA1D | calcium voltage-gated channel subunit alpha1 D |
|  | ENSG00000142408 |  | CACNG8 | calcium voltage-gated channel auxiliary subunit gamma 8 |
|  | ENSG00000004948 |  | CALCR | calcitonin receptor |
|  | ENSG00000001626 |  | CFTR | cystic fibrosis transmembrane conductance regulator |
|  | ENSG00000116254 |  | CHD5 | chromodomain helicase DNA binding protein 5 |
|  | ENSG00000204291 | D | COL15A1 | collagen type XV alpha 1 chain |
|  | ENSG00000204291 | D | COL15A1 | collagen type XV alpha 1 chain |
|  | ENSG00000182871 |  | COL18A1 | collagen type XVIII alpha 1 chain |
|  | ENSG00000130635 |  | COL5A1 | collagen type V alpha 1 chain |
|  | ENSG00000142173 |  | COL6A2 | collagen type VI alpha 2 chain |
|  | ENSG00000106789 |  | CORO2A | coronin 2A |
|  | ENSG00000215908 |  | CROCCP2 | ciliary rootlet coiled-coil, rootletin pseudogene 2 |
|  | ENSG00000244752 |  | CRYBB2 | crystallin beta B2 |
|  | ENSG00000107611 | D | CUBN | cubilin |
|  | ENSG00000107611 | D | CUBN | cubilin |
|  | ENSG00000261593 |  | CYP4A27P | cytochrome P450 family 4 subfamily A member 27, pseudogene |
|  | ENSG00000198010 |  | DLGAP2 | DLG associated protein 2 |
|  | ENSG00000152592 |  | DMP1 | dentin matrix acidic phosphoprotein 1 |
|  | ENSG00000177103 |  | DSCAML1 | DS cell adhesion molecule like 1 |
|  | ENSG00000046604 |  | DSG2 | desmoglein 2 |
|  | ENSG00000188716 |  | DUPD1 | dual specificity phosphatase and pro isomerase domain containing 1 |
|  | ENSG00000095203 |  | EPB41L4B | erythrocyte membrane protein band 4.1 like 4B |
|  | ENSG00000145242 |  | EPHA5 | EPH receptor A5 |
|  | ENSG00000112685 |  | EXOC2 | exocyst complex component 2 |
|  | ENSG00000100376 |  | FAM118A | family with sequence similarity 118 member A |
|  | ENSG00000174137 |  | FAM53A | family with sequence similarity 53 member A |
|  | ENSG00000163520 |  | FBLN2 | fibulin 2 |
|  | ENSG00000165140 |  | FBP1 | fructose-bisphosphatase 1 |
|  | ENSG00000073712 |  | FERMT2 | fermitin family member 2 |
|  | ENSG00000068078 |  | FGFR3 | fibroblast growth factor receptor 3 |
|  | ENSG00000112367 |  | FIG4 | FIG4 phosphoinositide 5-phosphatase |
|  | ENSG00000128591 |  | FLNC | filamin C |
|  | ENSG00000115414 |  | FN1 | fibronectin 1 |
|  | ENSG00000164694 |  | FNDC1 | fibronectin type III domain containing 1 |
|  | ENSG00000089154 |  | GCN1 | GCN1, eIF2 alpha kinase activator homolog |
|  | ENSG00000267486 |  | GLUD1P4 | glutamate dehydrogenase 1 pseudogene 4 |
|  | ENSG00000160360 |  | GPSM1 | G protein signaling modulator 1 |
|  | ENSG00000084754 |  | HADHA | hydroxyacyl-CoA dehydrogenase/3-ketoacyl-CoA thiolase/enoyl-CoA hydratase (trifunctional protein), alpha subunit |
|  | ENSG00000126107 | D | HECTD3 | HECT domain E3 ubiquitin protein ligase 3 |
|  | ENSG00000126107 | D | HECTD3 | HECT domain E3 ubiquitin protein ligase 3 |
|  | ENSG00000148357 |  | HMCN2 | hemicentin 2 |
|  | ENSG00000142798 |  | HSPG2 | heparan sulfate proteoglycan 2 |
|  | ENSG00000197081 | D | IGF2R | insulin like growth factor 2 receptor |
|  | ENSG00000197081 | D | IGF2R | insulin like growth factor 2 receptor |
|  | ENSG00000253265 |  | IGKV2-14 | immunoglobulin kappa variable 2-14 (pseudogene) |
|  | ENSG00000253998 |  | IGKV2-29 | immunoglobulin kappa variable 2-29 (gene/pseudogene) |
|  | ENSG00000253786 |  | IGLV3-15 | immunoglobulin lambda variable 3-15 (pseudogene) |
|  | ENSG00000113141 |  | IK | IK cytokine, down-regulator of HLA II |
|  | ENSG00000205339 |  | IPO7 | importin 7 |
|  | ENSG00000140575 |  | IQGAP1 | IQ motif containing GTPase activating protein 1 |
|  | ENSG00000156886 |  | ITGAD | integrin subunit alpha D |
|  | ENSG00000139626 |  | ITGB7 | integrin subunit beta 7 |
|  | ENSG00000096433 |  | ITPR3 | inositol 1,4,5-trisphosphate receptor type 3 |
|  | ENSG00000167548 |  | KMT2D | lysine methyltransferase 2D |
|  | ENSG00000198945 |  | L3MBTL3 | l(3)mbt-like 3 (Drosophila) |
|  | ENSG00000196569 |  | LAMA2 | laminin subunit alpha 2 |
|  | ENSG00000100079 |  | LGALS2 | galectin 2 |
|  | ENSG00000128342 |  | LIF | LIF, interleukin 6 family cytokine |
|  | ENSG00000223768 |  | LINC00205 | long intergenic non-protein coding RNA 205 |
|  | ENSG00000226521 |  | LOC100287072 | ribosomal protein S6 kinase B1 pseudogene |
|  | ENSG00000138095 |  | LRPPRC | leucine rich pentatricopeptide repeat containing |
|  | ENSG00000176809 |  | LRRC37A3 (includes others) | leucine rich repeat containing 37 member A3 |
|  | ENSG00000177239 |  | MAN1B1 | mannosidase alpha class 1B member 1 |
|  | ENSG00000185090 |  | MANEAL | mannosidase endo-alpha like |
|  | ENSG00000147316 |  | MCPH1 | microcephalin 1 |
|  | ENSG00000141434 |  | MEP1B | meprin A subunit beta |
|  | ENSG00000133808 |  | MICALCL | MICAL C-terminal like |
|  | ENSG00000211563 |  | mir-3065 | microRNA 3065 |
|  | ENSG00000159055 |  | MIS18A | MIS18 kinetochore protein A |
|  | ENSG00000186715 |  | MST1L | macrophage stimulating 1 like |
|  | ENSG00000186301 |  | MST1P2 | macrophage stimulating 1 pseudogene 2 |
|  | ENSG00000198793 |  | MTOR | mechanistic target of rapamycin |
|  | ENSG00000221986 |  | MYBPHL | myosin binding protein H like |
|  | ENSG00000006788 |  | MYH13 | myosin heavy chain 13 |
|  | ENSG00000133020 |  | MYH8 | myosin heavy chain 8 |
|  | ENSG00000128641 |  | MYO1B | myosin IB |
|  | ENSG00000095777 |  | MYO3A | myosin IIIA |
|  | ENSG00000197535 |  | MYO5A | myosin VA |
|  | ENSG00000196586 |  | MYO6 | myosin VI |
|  | ENSG00000223875 |  | NBEAP3 | neurobeachin pseudogene 3 |
|  | ENSG00000135577 |  | NMBR | neuromedin B receptor |
|  | ENSG00000074771 |  | NOX3 | NADPH oxidase 3 |
|  | ENSG00000099250 |  | NRP1 | neuropilin 1 |
|  | ENSG00000165802 |  | NSMF | NMDA receptor synaptonuclear signaling and neuronal migration factor |
|  | ENSG00000072682 |  | P4HA2 | prolyl 4-hydroxylase subunit alpha 2 |
|  | ENSG00000135749 |  | PCNX2 | pecanex homolog 2 (Drosophila) |
|  | ENSG00000132326 |  | PER2 | period circadian clock 2 |
|  | ENSG00000197724 |  | PHF2 | PHD finger protein 2 |
|  | ENSG00000092621 |  | PHGDH | phosphoglycerate dehydrogenase |
|  | ENSG00000155846 |  | PPARGC1B | PPARG coactivator 1 beta |
|  | ENSG00000275052 |  | PPP4R3B | protein phosphatase 4 regulatory subunit 3B |
|  | ENSG00000146005 |  | PSD2 | pleckstrin and Sec7 domain containing 2 |
|  | ENSG00000142949 |  | PTPRF | protein tyrosine phosphatase, receptor type F |
|  | ENSG00000124839 |  | RAB17 | RAB17, member RAS oncogene family |
|  | ENSG00000177548 |  | RABEP2 | rabaptin, RAB GTPase binding effector protein 2 |
|  | ENSG00000091428 |  | RAPGEF4 | Rap guanine nucleotide exchange factor 4 |
|  | ENSG00000179051 |  | RCC2 | regulator of chromosome condensation 2 |
|  | ENSG00000049449 |  | RCN1 | reticulocalbin 1 |
|  | ENSG00000213741 |  | RPS29 | ribosomal protein S29 |
|  | ENSG00000231544 |  | RSL24D1P11 | ribosomal L24 domain containing 1 pseudogene 11 |
|  | ENSG00000145284 |  | SCD5 | stearoyl-CoA desaturase 5 |
|  | ENSG00000168356 |  | SCN11A | sodium voltage-gated channel alpha subunit 11 |
|  | ENSG00000153253 |  | SCN3A | sodium voltage-gated channel alpha subunit 3 |
|  | ENSG00000214491 |  | SEC14L6 | SEC14 like lipid binding 6 |
|  | ENSG00000100934 |  | SEC23A | Sec23 homolog A, coat complex II component |
|  | ENSG00000112902 |  | SEMA5A | semaphorin 5A |
|  | ENSG00000082684 |  | SEMA5B | semaphorin 5B |
|  | ENSG00000086475 |  | SEPHS1 | selenophosphate synthetase 1 |
|  | ENSG00000057149 |  | SERPINB3 | serpin family B member 3 |
|  | ENSG00000152217 |  | SETBP1 | SET binding protein 1 |
|  | ENSG00000170624 |  | SGCD | sarcoglycan delta |
|  | ENSG00000163082 |  | SGPP2 | sphingosine-1-phosphate phosphatase 2 |
|  | ENSG00000146477 |  | SLC22A3 | solute carrier family 22 member 3 |
|  | ENSG00000186198 |  | SLC51B | solute carrier family 51 beta subunit |
|  | ENSG00000100191 |  | SLC5A4 | solute carrier family 5 member 4 |
|  | ENSG00000125870 |  | SNRPB2 | small nuclear ribonucleoprotein polypeptide B2 |
|  | ENSG00000159082 |  | SYNJ1 | synaptojanin 1 |
|  | ENSG00000115183 |  | TANC1 | tetratricopeptide repeat, ankyrin repeat and coiled-coil containing 1 |
|  | ENSG00000173662 |  | TAS1R1 | taste 1 receptor member 1 |
|  | ENSG00000231445 |  | TIMM8AP1 | translocase of inner mitochondrial membrane 8A pseudogene 1 |
|  | ENSG00000187045 |  | TMPRSS6 | transmembrane protease, serine 6 |
|  | ENSG00000141655 |  | TNFRSF11A | TNF receptor superfamily member 11a |
|  | ENSG00000131747 |  | TOP2A | topoisomerase (DNA) II alpha |
|  | ENSG00000137364 |  | TPMT | thiopurine S-methyltransferase |
|  | ENSG00000223417 | D | TRIM49D1/TRIM49D2 | tripartite motif containing 49D1 |
|  | ENSG00000233802 | D | TRIM49D1/TRIM49D2 | tripartite motif containing 49D1 |
|  | ENSG00000158022 |  | TRIM63 | tripartite motif containing 63 |
|  | ENSG00000092439 |  | TRPM7 | transient receptor potential cation channel subfamily M member 7 |
|  | ENSG00000187688 |  | TRPV2 | transient receptor potential cation channel subfamily V member 2 |
|  | ENSG00000196367 |  | TRRAP | transformation/transcription domain associated protein |
|  | ENSG00000211460 |  | TSN | translin |
|  | ENSG00000223962 |  | UBBP3 | ubiquitin B pseudogene 3 |
|  | ENSG00000138629 |  | UBL7 | ubiquitin like 7 |
|  | ENSG00000109814 |  | UGDH | UDP-glucose 6-dehydrogenase |
|  | ENSG00000241119 |  | UGT1A7 (includes others) | UDP glucuronosyltransferase family 1 member A10 |
|  | ENSG00000215110 |  | UGT2B25P | UDP glucuronosyltransferase family 2 member B25, pseudogene |
|  | ENSG00000177398 |  | UMODL1 | uromodulin like 1 |
|  | ENSG00000188690 |  | UROS | uroporphyrinogen III synthase |
|  | ENSG00000158125 |  | XDH | xanthine dehydrogenase |
|  | ENSG00000083896 |  | YTHDC1 | YTH domain containing 1 |
|  | ENSG00000134744 |  | ZCCHC11 | zinc finger CCHC-type containing 11 |
|  | ENSG00000101493 |  | ZNF516 | zinc finger protein 516 |
|  | ENSG00000248830 |  | ZNF807 | zinc finger protein 807 |
| **Over-represented in group M** | ENSG00000256340 |  | ABCC6P1 | ATP binding cassette subfamily C member 6 pseudogene 1 |
|  | ENSG00000153294 |  | ADGRF4 | adhesion G protein-coupled receptor F4 |
|  | ENSG00000172493 |  | AFF1 | AF4/FMR2 family member 1 |
|  | ENSG00000106351 |  | AGFG2 | ArfGAP with FG repeats 2 |
|  | ENSG00000153107 |  | ANAPC1 | anaphase promoting complex subunit 1 |
|  | ENSG00000172014 |  | ANKRD20A4 (includes others) | ankyrin repeat domain 20 family member A4 |
|  | ENSG00000224309 |  | ANKRD30BP2 | ankyrin repeat domain 30B pseudogene 2 |
|  | ENSG00000066279 |  | ASPM | abnormal spindle microtubule assembly |
|  | ENSG00000075673 |  | ATP12A | ATPase H+/K+ transporting non-gastric alpha2 subunit |
|  | ENSG00000204463 |  | BAG6 | BCL2 associated athanogene 6 |
|  | ENSG00000114439 |  | BBX | BBX, HMG-box containing |
|  | ENSG00000153162 |  | BMP6 | bone morphogenetic protein 6 |
|  | ENSG00000258780 |  | BMS1P15 | BMS1, ribosome biogenesis factor pseudogene 15 |
|  | ENSG00000258684 |  | BMS1P16 | BMS1, ribosome biogenesis factor pseudogene 16 |
|  | ENSG00000186190 |  | BPIFB3 | BPI fold containing family B member 3 |
|  | ENSG00000120029 |  | C10orf76 | chromosome 10 open reading frame 76 |
|  | ENSG00000215595 |  | C20orf202 | chromosome 20 open reading frame 202 |
|  | ENSG00000224389 |  | C4A/C4B | complement C4B (Chido blood group) |
|  | ENSG00000198216 |  | CACNA1E | calcium voltage-gated channel subunit alpha1 E |
|  | ENSG00000081803 |  | CADPS2 | calcium dependent secretion activator 2 |
|  | ENSG00000064989 |  | CALCRL | calcitonin receptor like receptor |
|  | ENSG00000145386 |  | CCNA2 | cyclin A2 |
|  | ENSG00000122674 |  | CCZ1/CCZ1B | CCZ1 homolog B, vacuolar protein trafficking and biogenesis associated |
|  | ENSG00000196776 |  | CD47 | CD47 molecule |
|  | ENSG00000114013 |  | CD86 | CD86 molecule |
|  | ENSG00000139610 |  | CELA1 | chymotrypsin like elastase family member 1 |
|  | ENSG00000181800 |  | CELF2-AS1 | CELF2 antisense RNA 1 |
|  | ENSG00000166451 |  | CENPN | centromere protein N |
|  | ENSG00000100888 |  | CHD8 | chromodomain helicase DNA binding protein 8 |
|  | ENSG00000101421 |  | CHMP4B | charged multivesicular body protein 4B |
|  | ENSG00000054938 |  | CHRDL2 | chordin like 2 |
|  | ENSG00000188037 |  | CLCN1 | chloride voltage-gated channel 1 |
|  | ENSG00000155052 |  | CNTNAP5 | contactin associated protein like 5 |
|  | ENSG00000111799 |  | COL12A1 | collagen type XII alpha 1 chain |
|  | ENSG00000124749 |  | COL21A1 | collagen type XXI alpha 1 chain |
|  | ENSG00000169436 |  | COL22A1 | collagen type XXII alpha 1 chain |
|  | ENSG00000110090 |  | CPT1A | carnitine palmitoyltransferase 1A |
|  | ENSG00000143162 |  | CREG1 | cellular repressor of E1A stimulated genes 1 |
|  | ENSG00000121966 |  | CXCR4 | C-X-C motif chemokine receptor 4 |
|  | ENSG00000225082 |  | DAP3P1 | death associated protein 3 pseudogene 1 |
|  | ENSG00000139734 |  | DIAPH3 | diaphanous related formin 3 |
|  | ENSG00000083520 |  | DIS3 | DIS3 homolog, exosome endoribonuclease and 3'-5' exoribonuclease |
|  | ENSG00000197653 | D | DNAH10 | dynein axonemal heavy chain 10 |
|  | ENSG00000197653 | D | DNAH10 | dynein axonemal heavy chain 10 |
|  | ENSG00000105877 |  | DNAH11 | dynein axonemal heavy chain 11 |
|  | ENSG00000124721 |  | DNAH8 | dynein axonemal heavy chain 8 |
|  | ENSG00000232125 |  | DYTN | dystrotelin |
|  | ENSG00000116406 |  | EDEM3 | ER degradation enhancing alpha-mannosidase like protein 3 |
|  | ENSG00000249855 |  | EEF1A1P19 | eukaryotic translation elongation factor 1 alpha 1 pseudogene 19 |
|  | ENSG00000205609 |  | EIF3CL | eukaryotic translation initiation factor 3 subunit C like |
|  | ENSG00000149218 |  | ENDOD1 | endonuclease domain containing 1 |
|  | ENSG00000088367 |  | EPB41L1 | erythrocyte membrane protein band 4.1 like 1 |
|  | ENSG00000117868 |  | ESYT2 | extended synaptotagmin 2 |
|  | ENSG00000010030 |  | ETV7 | ETS variant 7 |
|  | ENSG00000188107 |  | EYS | eyes shut homolog (Drosophila) |
|  | ENSG00000175170 |  | FAM182B | family with sequence similarity 182 member B |
|  | ENSG00000277988 |  | FAM30B |  |
|  | ENSG00000259698 |  | FAM30C | family with sequence similarity 30 member C |
|  | ENSG00000214814 |  | FER1L6 | fer-1 like family member 6 |
|  | ENSG00000198225 |  | FKBP1C | FK506 binding protein 1C |
|  | ENSG00000102755 |  | FLT1 | fms related tyrosine kinase 1 |
|  | ENSG00000122025 | D | FLT3 | fms related tyrosine kinase 3 |
|  | ENSG00000122025 | D | FLT3 | fms related tyrosine kinase 3 |
|  | ENSG00000129245 |  | FXR2 | FMR1 autosomal homolog 2 |
|  | ENSG00000107862 |  | GBF1 | golgi brefeldin A resistant guanine nucleotide exchange factor 1 |
|  | ENSG00000136487 |  | GH2 | growth hormone 2 |
|  | ENSG00000118702 |  | GHRH | growth hormone releasing hormone |
|  | ENSG00000171115 |  | GIMAP8 | GTPase, IMAP family member 8 |
|  | ENSG00000104499 |  | GML | glycosylphosphatidylinositol anchored molecule like |
|  | ENSG00000214415 |  | GNAT3 | G protein subunit alpha transducin 3 |
|  | ENSG00000107937 |  | GTPBP4 | GTP binding protein 4 |
|  | ENSG00000276550 | D | HERC2P2 | hect domain and RLD 2 pseudogene 2 |
|  | ENSG00000276550 | D | HERC2P2 | hect domain and RLD 2 pseudogene 2 |
|  | ENSG00000276550 | D | HERC2P2 | hect domain and RLD 2 pseudogene 2 |
|  | ENSG00000276550 | D | HERC2P2 | hect domain and RLD 2 pseudogene 2 |
|  | ENSG00000276550 | D | HERC2P2 | hect domain and RLD 2 pseudogene 2 |
|  | ENSG00000180229 | D | HERC2P3 | hect domain and RLD 2 pseudogene 3 |
|  | ENSG00000180229 | D | HERC2P3 | hect domain and RLD 2 pseudogene 3 |
|  | ENSG00000206149 |  | HERC2P9 | hect domain and RLD 2 pseudogene 9 |
|  | ENSG00000275713 |  | HIST1H2BH | histone cluster 1 H2B family member h |
|  | ENSG00000143341 |  | HMCN1 | hemicentin 1 |
|  | ENSG00000164749 |  | HNF4G | hepatocyte nuclear factor 4 gamma |
|  | ENSG00000205412 |  | HNRNPA1P20 | heterogeneous nuclear ribonucleoprotein A1 pseudogene 20 |
|  | ENSG00000205100 |  | HSP90AA4P | heat shock protein 90 alpha family class A member 4, pseudogene |
|  | ENSG00000270824 |  | IGHD5OR15-5B | immunoglobulin heavy diversity 5/OR15-5B (non-functional) |
|  | ENSG00000270685 |  | IGHV1OR15-6 | immunoglobulin heavy variable 1/OR15-6 (pseudogene) |
|  | ENSG00000104998 |  | IL27RA | interleukin 27 receptor subunit alpha |
|  | ENSG00000136696 |  | IL36B | interleukin 36, beta |
|  | ENSG00000104331 |  | IMPAD1 | inositol monophosphatase domain containing 1 |
|  | ENSG00000120645 |  | IQSEC3 | IQ motif and Sec7 domain 3 |
|  | ENSG00000150995 |  | ITPR1 | inositol 1,4,5-trisphosphate receptor type 1 |
|  | ENSG00000105639 |  | JAK3 | Janus kinase 3 |
|  | ENSG00000118193 |  | KIF14 | kinesin family member 14 |
|  | ENSG00000242019 |  | KIR3DL3 | killer cell immunoglobulin like receptor, three Ig domains and long cytoplasmic tail 3 |
|  | ENSG00000250563 |  | KNOP1P5 | lysine rich nucleolar protein 1 pseudogene 5 |
|  | ENSG00000167916 |  | KRT24 | keratin 24 |
|  | ENSG00000091136 |  | LAMB1 | laminin subunit beta 1 |
|  | ENSG00000174106 |  | LEMD3 | LEM domain containing 3 |
|  | ENSG00000250979 |  | LOC392232 | transient receptor potential cation channel subfamily A member 1 pseudogene |
|  | ENSG00000108829 |  | LRRC59 | leucine rich repeat containing 59 |
|  | ENSG00000188906 |  | LRRK2 | leucine rich repeat kinase 2 |
|  | ENSG00000149657 |  | LSM14B | LSM family member 14B |
|  | ENSG00000140943 |  | MBTPS1 | membrane bound transcription factor peptidase, site 1 |
|  | ENSG00000101898 |  | MCTS2P | malignant T-cell amplified sequence 2, pseudogene |
|  | ENSG00000136146 |  | MED4 | mediator complex subunit 4 |
|  | ENSG00000222831 |  | mir-1537 | microRNA 1537 |
|  | ENSG00000207838 |  | mir-515 | microRNA 520c |
|  | ENSG00000207974 |  | mir-557 | microRNA 557 |
|  | ENSG00000101353 |  | MROH8 | maestro heat like repeat family member 8 |
|  | ENSG00000211459 |  | MT-RNR1 | s-rRNA |
|  | ENSG00000210082 |  | MT-RNR2 | l-rRNA |
|  | ENSG00000085760 |  | MTIF2 | mitochondrial translational initiation factor 2 |
|  | ENSG00000139505 | D | MTMR6 | myotubularin related protein 6 |
|  | ENSG00000139505 | D | MTMR6 | myotubularin related protein 6 |
|  | ENSG00000139505 | D | MTMR6 | myotubularin related protein 6 |
|  | ENSG00000198793 |  | MTOR | mechanistic target of rapamycin |
|  | ENSG00000120662 |  | MTRF1 | mitochondrial translation release factor 1 |
|  | ENSG00000205592 | D | MUC19 | mucin 19, oligomeric |
|  | ENSG00000205592 | D | MUC19 | mucin 19, oligomeric |
|  | ENSG00000005810 |  | MYCBP2 | MYC binding protein 2, E3 ubiquitin protein ligase |
|  | ENSG00000144821 |  | MYH15 | myosin heavy chain 15 |
|  | ENSG00000078814 |  | MYH7B | myosin heavy chain 7B |
|  | ENSG00000099331 |  | MYO9B | myosin IXB |
|  | ENSG00000111704 |  | NANOG | Nanog homeobox |
|  | ENSG00000172915 |  | NBEA | neurobeachin |
|  | ENSG00000136937 |  | NCBP1 | nuclear cap binding protein subunit 1 |
|  | ENSG00000124151 |  | NCOA3 | nuclear receptor coactivator 3 |
|  | ENSG00000183091 |  | NEB | nebulin |
|  | ENSG00000270831 | D | NF1P1 | neurofibromin 1 pseudogene 1 |
|  | ENSG00000270831 | D | NF1P1 | neurofibromin 1 pseudogene 1 |
|  | ENSG00000258997 |  | NF1P2 | neurofibromin 1 pseudogene 2 |
|  | ENSG00000148200 |  | NR6A1 | nuclear receptor subfamily 6 group A member 1 |
|  | ENSG00000126952 |  | NXF5 | nuclear RNA export factor 5 |
|  | ENSG00000155087 |  | ODF1 | outer dense fiber of sperm tails 1 |
|  | ENSG00000187950 |  | OVCH1 | ovochymase 1 |
|  | ENSG00000184588 |  | PDE4B | phosphodiesterase 4B |
|  | ENSG00000146247 |  | PHIP | pleckstrin homology domain interacting protein |
|  | ENSG00000277481 |  | PKD1L3 | polycystin 1 like 3, transient receptor potential channel interacting |
|  | ENSG00000205038 |  | PKHD1L1 | PKHD1 like 1 |
|  | ENSG00000114805 |  | PLCH1 | phospholipase C eta 1 |
|  | ENSG00000178209 |  | PLEC | plectin |
|  | ENSG00000203805 |  | PLPP4 | phospholipid phosphatase 4 |
|  | ENSG00000196313 |  | POM121/POM121C | POM121 transmembrane nucleoporin |
|  | ENSG00000133110 |  | POSTN | periostin |
|  | ENSG00000230031 |  | POTEB/POTEB2 | POTE ankyrin domain family member B2 |
|  | ENSG00000275052 |  | PPP4R3B | protein phosphatase 4 regulatory subunit 3B |
|  | ENSG00000165630 |  | PRPF18 | pre-mRNA processing factor 18 |
|  | ENSG00000136875 |  | PRPF4 | pre-mRNA processing factor 4 |
|  | ENSG00000108344 |  | PSMD3 | proteasome 26S subunit, non-ATPase 3 |
|  | ENSG00000127947 |  | PTPN12 | protein tyrosine phosphatase, non-receptor type 12 |
|  | ENSG00000153707 |  | PTPRD | protein tyrosine phosphatase, receptor type D |
|  | ENSG00000068976 |  | PYGM | glycogen phosphorylase, muscle associated |
|  | ENSG00000170471 |  | RALGAPB | Ral GTPase activating protein non-catalytic beta subunit |
|  | ENSG00000237207 |  | RBM17P3 | RNA binding motif protein 17 pseudogene 3 |
|  | ENSG00000143344 |  | RGL1 | ral guanine nucleotide dissociation stimulator like 1 |
|  | ENSG00000112077 |  | RHAG | Rh-associated glycoprotein |
|  | ENSG00000132972 |  | RNF17 | ring finger protein 17 |
|  | ENSG00000111832 |  | RWDD1 | RWD domain containing 1 |
|  | ENSG00000198626 |  | RYR2 | ryanodine receptor 2 |
|  | ENSG00000129657 |  | SEC14L1 | SEC14 like lipid binding 1 |
|  | ENSG00000103184 |  | SEC14L5 | SEC14 like lipid binding 5 |
|  | ENSG00000197860 |  | SGTB | small glutamine rich tetratricopeptide repeat containing beta |
|  | ENSG00000158296 |  | SLC13A3 | solute carrier family 13 member 3 |
|  | ENSG00000163406 |  | SLC15A2 | solute carrier family 15 member 2 |
|  | ENSG00000091137 |  | SLC26A4 | solute carrier family 26 member 4 |
|  | ENSG00000181804 |  | SLC9A9 | solute carrier family 9 member A9 |
|  | ENSG00000072501 |  | SMC1A | structural maintenance of chromosomes 1A |
|  | ENSG00000223979 |  | SMCR2 | Smith-Magenis syndrome chromosome region, candidate 2 (non-protein coding) |
|  | ENSG00000198952 |  | SMG5 | SMG5, nonsense mediated mRNA decay factor |
|  | ENSG00000115904 |  | SOS1 | SOS Ras/Rac guanine nucleotide exchange factor 1 |
|  | ENSG00000185591 |  | SP1 | Sp1 transcription factor |
|  | ENSG00000104450 |  | SPAG1 | sperm associated antigen 1 |
|  | ENSG00000274279 |  | SPATA31E3P | SPATA31 subfamily E member 3, pseudogene |
|  | ENSG00000196369 |  | SRGAP2B | SLIT-ROBO Rho GTPase activating protein 2B |
|  | ENSG00000174780 |  | SRP72 | signal recognition particle 72 |
|  | ENSG00000196228 |  | SULT1C3 | sulfotransferase family 1C member 3 |
|  | ENSG00000136111 |  | TBC1D4 | TBC1 domain family member 4 |
|  | ENSG00000137076 |  | TLN1 | talin 1 |
|  | ENSG00000137747 |  | TMPRSS13 | transmembrane protease, serine 13 |
|  | ENSG00000248290 |  | TNXA | tenascin XA (pseudogene) |
|  | ENSG00000067369 |  | TP53BP1 | tumor protein p53 binding protein 1 |
|  | ENSG00000165914 |  | TTC7B | tetratricopeptide repeat domain 7B |
|  | ENSG00000155657 |  | TTN | titin |
|  | ENSG00000227551 | D | USP17L24 (includes others) | ubiquitin specific peptidase 17-like family member 24 |
|  | ENSG00000231051 | D | USP17L24 (includes others) | ubiquitin specific peptidase 17-like family member 24 |
|  | ENSG00000132952 |  | USPL1 | ubiquitin specific peptidase like 1 |
|  | ENSG00000152818 |  | UTRN | utrophin |
|  | ENSG00000189068 |  | VSTM1 | V-set and transmembrane domain containing 1 |
|  | ENSG00000164961 |  | WASHC5 | WASH complex subunit 5 |
|  | ENSG00000163625 |  | WDFY3 | WD repeat and FYVE domain containing 3 |
|  | ENSG00000124535 |  | WRNIP1 | Werner helicase interacting protein 1 |
|  | ENSG00000132953 |  | XPO4 | exportin 4 |
|  | ENSG00000146858 |  | ZC3HAV1L | zinc finger CCCH-type containing, antiviral 1 like |
|  | ENSG00000196867 |  | ZFP28 | ZFP28 zinc finger protein |
|  | ENSG00000223614 |  | ZNF735 | zinc finger protein 735 |

**Supporting Table 3:** Verification test. A Random Forest classification was obtained after 100 iterations, extracting two randomly selected samples from each group, metastatic (M) and non-metastatic (N), and generating a predictive model, with the 16 remaining samples (eight per group) as a training set. A verification test was performed to confirm that the algorithm was able to classify extracted samples into their corresponding groups of origin. The average probabilities obtained for belonging to one group or another are shown.

|  | **Average probability obtained for belonging to group M** | **Average probability obtained for belonging to group N** |
| --- | --- | --- |
| Extracted sample #1 from group M | 0.68 | 0.32 |
| Extracted sample #2 from group M | 0.67 | 0.33 |
| Extracted sample #1 from group N | 0.36 | 0.64 |
| Extracted sample #2 from group N | 0.35 | 0.65 |

**Supporting Table 4:** Classification of unclassifiable (U) patients by the algorithm. The obtained probabilities for each patient from group U of belonging to the metastatic (M) or non-metastatic (N) group using the predictive algorithm are shown.

| **Patient ID** | **Obtained probability for belonging to group M** | **Obtained probability for belonging to group N** |
| --- | --- | --- |
| p04 | 0.60 | 0.40 |
| p08 | 0.53 | 0.47 |
| p12 | 0.48 | 0.52 |
| p15 | 0.55 | 0.45 |
| p23 | 0.56 | 0.44 |
| p28 | 0.55 | 0.45 |
| p57 | 0.49 | 0.51 |
| p58 | 0.51 | 0.49 |
| p63 | 0.60 | 0.40 |
| p66 | 0.51 | 0.49 |

**Supporting Table 5:** Complete list of main IPA function categories affected by over-present genes in group M with their associated range of p-values. The number of genes involved in each category is shown.

| **Main functions categories in Group M** | | |
| --- | --- | --- |
| **Function** | **Number of genes** | **p-value** |
| Auditory and Vestibular System Development and Function | 1 | 7,91E-03-7,91E-03 |
| Auditory Disease | 3 | 6,13E-04-7,91E-03 |
| Cancer | 146 | 1,84E-09-8,76E-03 |
| Carbohydrate Metabolism | 1 | 7,91E-03-7,91E-03 |
| Cardiovascular Disease | 11 | 2,16E-03-7,91E-03 |
| Cardiovascular System Development and Function | 17 | 2,97E-05-8,76E-03 |
| Cell Cycle | 13 | 2,69E-03-8,76E-03 |
| Cell Death and Survival | 15 | 1,83E-03-8,76E-03 |
| Cell Morphology | 21 | 1,69E-03-8,04E-03 |
| Cell Signaling | 13 | 1,76E-03-8,76E-03 |
| Cell-mediated Immune Response | 5 | 4,76E-03-8,76E-03 |
| Cell-To-Cell Signaling and Interaction | 13 | 4,16E-03-8,76E-03 |
| Cellular Assembly and Organization | 8 | 9,14E-04-7,91E-03 |
| Cellular Compromise | 6 | 7,91E-03-7,91E-03 |
| Cellular Development | 15 | 2,97E-05-7,91E-03 |
| Cellular Function and Maintenance | 30 | 3,7E-04-7,91E-03 |
| Cellular Growth and Proliferation | 12 | 2,97E-05-7,91E-03 |
| Cellular Movement | 16 | 1,86E-04-8,21E-03 |
| Connective Tissue Development and Function | 6 | 3,9E-03-7,91E-03 |
| Connective Tissue Disorders | 21 | 4,29E-04-7,91E-03 |
| Dermatological Diseases and Conditions | 111 | 1,09E-08-8,76E-03 |
| Developmental Disorder | 30 | 1,09E-03-7,91E-03 |
| Digestive System Development and Function | 1 | 7,91E-03-7,91E-03 |
| DNA Replication, Recombination, and Repair | 2 | 7,91E-03-7,91E-03 |
| Embryonic Development | 29 | 2,97E-05-7,91E-03 |
| Endocrine System Development and Function | 3 | 3,88E-03-7,91E-03 |
| Endocrine System Disorders | 70 | 8,93E-07-7,91E-03 |
| Gastrointestinal Disease | 131 | 8,93E-07-8,76E-03 |
| Gene Expression | 8 | 7,69E-03-7,91E-03 |
| Hematological Disease | 51 | 8,36E-06-8,09E-03 |
| Hematological System Development and Function | 14 | 3,7E-04-8,76E-03 |
| Hematopoiesis | 5 | 3,7E-04-7,91E-03 |
| Hepatic System Disease | 80 | 1,14E-05-8,76E-03 |
| Hereditary Disorder | 36 | 2,69E-03-7,91E-03 |
| Humoral Immune Response | 7 | 1,69E-03-7,91E-03 |
| Immune Cell Trafficking | 7 | 4,76E-03-8,29E-03 |
| Immunological Disease | 38 | 2,55E-05-8,09E-03 |
| Infectious Diseases | 2 | 7,91E-03-7,91E-03 |
| Inflammatory Disease | 6 | 5,92E-03-6,11E-03 |
| Inflammatory Response | 14 | 5,92E-03-8,76E-03 |
| Lipid Metabolism | 1 | 7,91E-03-7,91E-03 |
| Lymphoid Tissue Structure and Development | 7 | 3,7E-04-7,91E-03 |
| Metabolic Disease | 9 | 4,45E-03-7,91E-03 |
| Molecular Transport | 17 | 2,69E-03-8,76E-03 |
| Nervous System Development and Function | 8 | 6,11E-03-7,91E-03 |
| Neurological Disease | 41 | 2,72E-07-7,91E-03 |
| Nucleic Acid Metabolism | 9 | 2,69E-03-8,38E-03 |
| Ophthalmic Disease | 3 | 7,91E-03-7,91E-03 |
| Organ Development | 16 | 2,97E-05-7,91E-03 |
| Organ Morphology | 7 | 7,91E-03-7,91E-03 |
| Organismal Development | 37 | 2,97E-05-8,76E-03 |
| Organismal Functions | 3 | 7,91E-03-7,91E-03 |
| Organismal Injury and Abnormalities | 147 | 1,84E-09-8,76E-03 |
| Protein Synthesis | 3 | 3,88E-03-3,88E-03 |
| Psychological Disorders | 6 | 1,09E-03-7,91E-03 |
| Renal and Urological Disease | 30 | 8,03E-05-8,47E-03 |
| Reproductive System Development and Function | 2 | 7,91E-03-7,91E-03 |
| Reproductive System Disease | 103 | 1,84E-09-8,71E-03 |
| Respiratory Disease | 49 | 7,72E-08-7,91E-03 |
| RNA Trafficking | 3 | 4,45E-03-4,45E-03 |
| Skeletal and Muscular Disorders | 39 | 4,29E-04-7,91E-03 |
| Skeletal and Muscular System Development and Function | 19 | 1,15E-04-7,91E-03 |
| Small Molecule Biochemistry | 11 | 2,69E-03-8,38E-03 |
| Tissue Development | 21 | 2,97E-05-8,29E-03 |
| Tissue Morphology | 12 | 3,65E-03-8,76E-03 |
| Tumor Morphology | 12 | 7,66E-05-8,76E-03 |
| Vitamin and Mineral Metabolism | 3 | 7,91E-03-8,76E-03 |

**Supporting Table 6:** Complete list of main IPA function categories affected by over-present genes in group N with their associated range of p-values. The number of genes involved in each category is shown.

| **Main functions categories in Group N** | | |
| --- | --- | --- |
| **Function** | **Number of genes** | **p-value** |
| Amino Acid Metabolism | 3 | 4,91E-03-4,91E-03 |
| Antimicrobial Response | 1 | 6,48E-03-6,48E-03 |
| Auditory and Vestibular System Development and Function | 5 | 4,65E-03-4,65E-03 |
| Auditory Disease | 4 | 1,62E-03-6,48E-03 |
| Behavior | 10 | 3,98E-03-3,98E-03 |
| Cancer | 120 | 5,47E-10-6,48E-03 |
| Carbohydrate Metabolism | 5 | 6,13E-04-6,48E-03 |
| Cardiovascular Disease | 24 | 7,91E-05-6,48E-03 |
| Cardiovascular System Development and Function | 27 | 7,91E-05-6,48E-03 |
| Cell Cycle | 3 | 6,48E-03-6,48E-03 |
| Cell Death and Survival | 5 | 3,1E-03-6,48E-03 |
| Cell Morphology | 41 | 9,16E-05-6,48E-03 |
| Cell Signaling | 6 | 5,68E-03-5,68E-03 |
| Cell-mediated Immune Response | 1 | 6,48E-03-6,48E-03 |
| Cell-To-Cell Signaling and Interaction | 31 | 2,27E-04-6,48E-03 |
| Cellular Assembly and Organization | 40 | 1,4E-04-6,48E-03 |
| Cellular Compromise | 6 | 4,16E-05-6,48E-03 |
| Cellular Development | 31 | 3,23E-06-6,48E-03 |
| Cellular Function and Maintenance | 41 | 1,4E-04-5,35E-03 |
| Cellular Growth and Proliferation | 29 | 3,23E-06-6,48E-03 |
| Cellular Movement | 42 | 5,66E-06-6,48E-03 |
| Connective Tissue Development and Function | 19 | 1,24E-04-6,48E-03 |
| Connective Tissue Disorders | 18 | 1,16E-04-6,48E-03 |
| Dermatological Diseases and Conditions | 90 | 4,57E-08-6,48E-03 |
| Developmental Disorder | 24 | 1,9E-03-6,48E-03 |
| Digestive System Development and Function | 23 | 8,27E-04-6,48E-03 |
| DNA Replication, Recombination, and Repair | 2 | 4,7E-03-6,48E-03 |
| Drug Metabolism | 2 | 4,16E-05-6,48E-03 |
| Embryonic Development | 39 | 1,3E-05-6,48E-03 |
| Endocrine System Development and Function | 5 | 6,47E-03-6,48E-03 |
| Endocrine System Disorders | 55 | 1,51E-05-6,48E-03 |
| Gastrointestinal Disease | 115 | 5,47E-10-6,48E-03 |
| Hematological Disease | 40 | 7E-04-6,48E-03 |
| Hematological System Development and Function | 8 | 5,91E-03-6,48E-03 |
| Hematopoiesis | 7 | 5,91E-03-6,48E-03 |
| Hepatic System Development and Function | 8 | 8,27E-04-3,02E-03 |
| Hepatic System Disease | 66 | 2,79E-05-5,95E-03 |
| Hereditary Disorder | 45 | 1,71E-04-6,48E-03 |
| Immunological Disease | 38 | 7E-04-6,48E-03 |
| Infectious Diseases | 2 | 6,48E-03-6,48E-03 |
| Inflammatory Disease | 8 | 6,25E-03-6,48E-03 |
| Inflammatory Response | 8 | 6,25E-03-6,48E-03 |
| Lipid Metabolism | 5 | 6,13E-04-6,48E-03 |
| Lymphoid Tissue Structure and Development | 7 | 5,91E-03-6,48E-03 |
| Metabolic Disease | 18 | 3,83E-05-6,48E-03 |
| Molecular Transport | 32 | 4,61E-04-6,48E-03 |
| Nervous System Development and Function | 33 | 3,23E-06-6,48E-03 |
| Neurological Disease | 49 | 4,16E-05-6,48E-03 |
| Nucleic Acid Metabolism | 4 | 4,16E-05-6,48E-03 |
| Nutritional Disease | 10 | 1,75E-03-6,48E-03 |
| Ophthalmic Disease | 7 | 4,16E-05-6,48E-03 |
| Organ Development | 34 | 9,16E-05-6,48E-03 |
| Organ Morphology | 29 | 9,16E-05-6,48E-03 |
| Organismal Development | 55 | 1,3E-05-6,48E-03 |
| Organismal Injury and Abnormalities | 120 | 5,47E-10-6,48E-03 |
| Organismal Survival | 44 | 4,12E-06-4,78E-03 |
| Protein Synthesis | 5 | 1,63E-03-1,63E-03 |
| Protein Trafficking | 11 | 1,42E-03-6,48E-03 |
| Psychological Disorders | 1 | 6,48E-03-6,48E-03 |
| Renal and Urological Disease | 15 | 4,75E-04-3,88E-03 |
| Renal and Urological System Development and Function | 1 | 6,48E-03-6,48E-03 |
| Reproductive System Development and Function | 13 | 9E-04-6,48E-03 |
| Reproductive System Disease | 80 | 4,65E-09-6,48E-03 |
| Respiratory Disease | 42 | 3,47E-05-6,48E-03 |
| Respiratory System Development and Function | 13 | 3,47E-05-2,2E-03 |
| Skeletal and Muscular Disorders | 47 | 9,57E-05-6,48E-03 |
| Skeletal and Muscular System Development and Function | 22 | 1,24E-04-6,48E-03 |
| Small Molecule Biochemistry | 14 | 4,16E-05-6,48E-03 |
| Tissue Development | 48 | 3,23E-06-6,48E-03 |
| Tissue Morphology | 40 | 9,16E-05-6,48E-03 |
| Tumor Morphology | 6 | 7,72E-04-6,48E-03 |
| Visual System Development and Function | 10 | 9,16E-05-4,71E-03 |
| Vitamin and Mineral Metabolism | 6 | 5,68E-03-5,68E-03 |

**Supporting Table 7:** Complete list of IPA networks in which over-present genes in group M are involved with their associated scores (based on the number of over represented genes in the network with respect to the global size of that network). These networks are associated with certain diseases and functions shown in the table. The number of genes involved in each network is also shown.

| **ID** | **Score** | **Number of Molecules** | **Top Diseases and Functions** |
| --- | --- | --- | --- |
| 1 | 39 | 22 | Cardiovascular System Development and Function, Embryonic Development, Organ Development |
| 2 | 30 | 18 | Cancer, Connective Tissue Disorders, Organismal Injury and Abnormalities |
| 3 | 30 | 18 | Cancer, Endocrine System Disorders, Gastrointestinal Disease |
| 4 | 28 | 17 | Cellular Movement, Cell-mediated Immune Response, Hematological System Development and Function |
| 5 | 21 | 14 | Cell Cycle, Developmental Disorder, Hereditary Disorder |
| 6 | 21 | 14 | Cellular Development, Embryonic Development, Organismal Development |
| 7 | 17 | 12 | Cellular Assembly and Organization, Cell Morphology, Cellular Development |
| 8 | 17 | 12 | Organ Morphology, Reproductive System Development and Function, Cancer |
| 9 | 17 | 12 | Cell Cycle, Cancer, Organismal Injury and Abnormalities |
| 10 | 9 | 7 | Post-Translational Modification, Developmental Disorder, Hereditary Disorder |
| 11 | 2 | 1 | Cardiovascular System Development and Function, Cell Cycle, Embryonic Development |
| 12 | 2 | 1 | Inflammatory Disease, Inflammatory Response, Organismal Injury and Abnormalities |
| 13 | 2 | 1 | Cell Cycle, Cellular Development, Developmental Disorder |

**Supporting Table 8:** Complete list of IPA networks in which over-present genes in group N are involved with their associated scores (based on the number of over represented genes in the network with respect to the global size of that network). These networks are associated with certain diseases and functions shown in the table. The number of genes involved in each network is also shown.

| **ID** | **Score** | **Number of Molecules** | **Top Diseases and Functions** |
| --- | --- | --- | --- |
| 1 | 46 | 24 | Cellular Assembly and Organization, Cellular Function and Maintenance, Cellular Movement |
| 2 | 36 | 20 | Cancer, Connective Tissue Disorders, Organismal Injury and Abnormalities |
| 3 | 36 | 20 | Hematological Disease, Immunological Disease, Tissue Development |
| 4 | 20 | 13 | Digestive System Development and Function, Embryonic Development, Hepatic System Development and Function |
| 5 | 16 | 11 | Lipid Metabolism, Small Molecule Biochemistry, Carbohydrate Metabolism |
| 6 | 16 | 11 | Connective Tissue Disorders, Inflammatory Disease, Organismal Injury and Abnormalities |
| 7 | 16 | 11 | Cell-To-Cell Signaling and Interaction, Small Molecule Biochemistry, Cell Death and Survival |
| 8 | 11 | 8 | Cellular Development, Cellular Growth and Proliferation, Endocrine System Development and Function |
| 9 | 7 | 6 | Carbohydrate Metabolism, Energy Production, Nucleic Acid Metabolism |
| 10 | 2 | 1 | Cell Morphology, Cellular Assembly and Organization, Cellular Compromise |
| 11 | 2 | 1 |  |

**Supporting Figure 1:** Complete list of 56 specific pathways from PANTHER public database (identified by PANTHER specific codes) in which 139 genes (to which differentially present exons belong both from groups M and N) are classified.


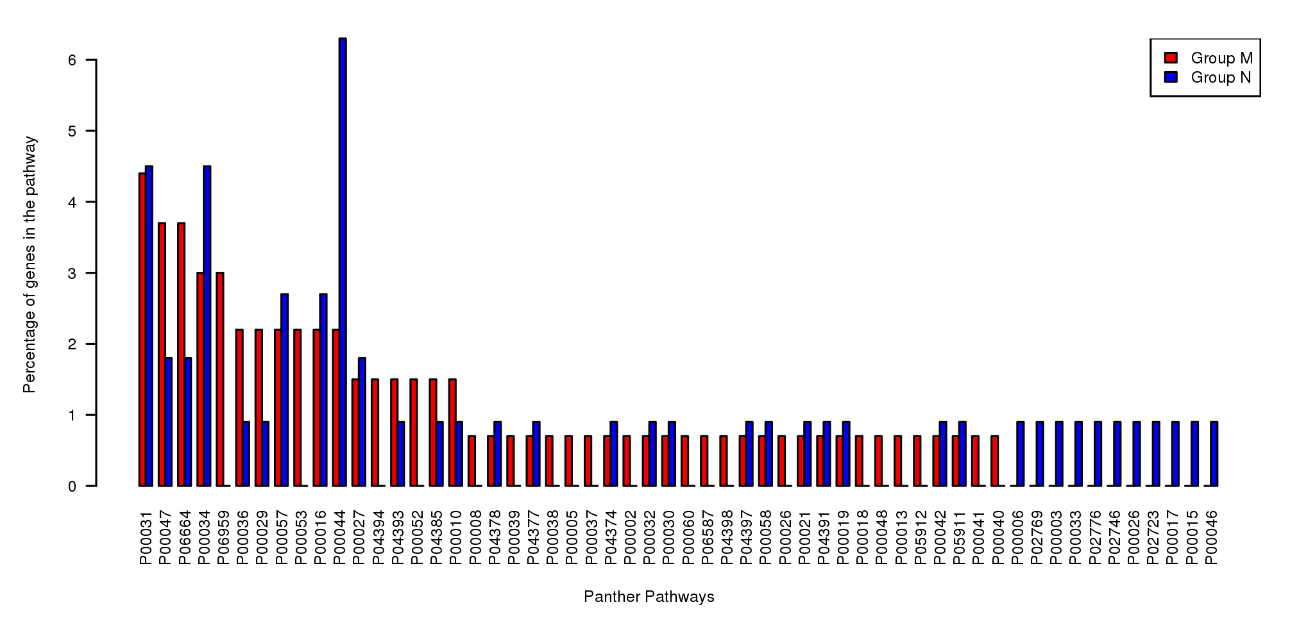


**Supporting Figure 2:** Complete list of main function categories from IPA. Functions are listed in descending other by the percentage of genes involved in each category.


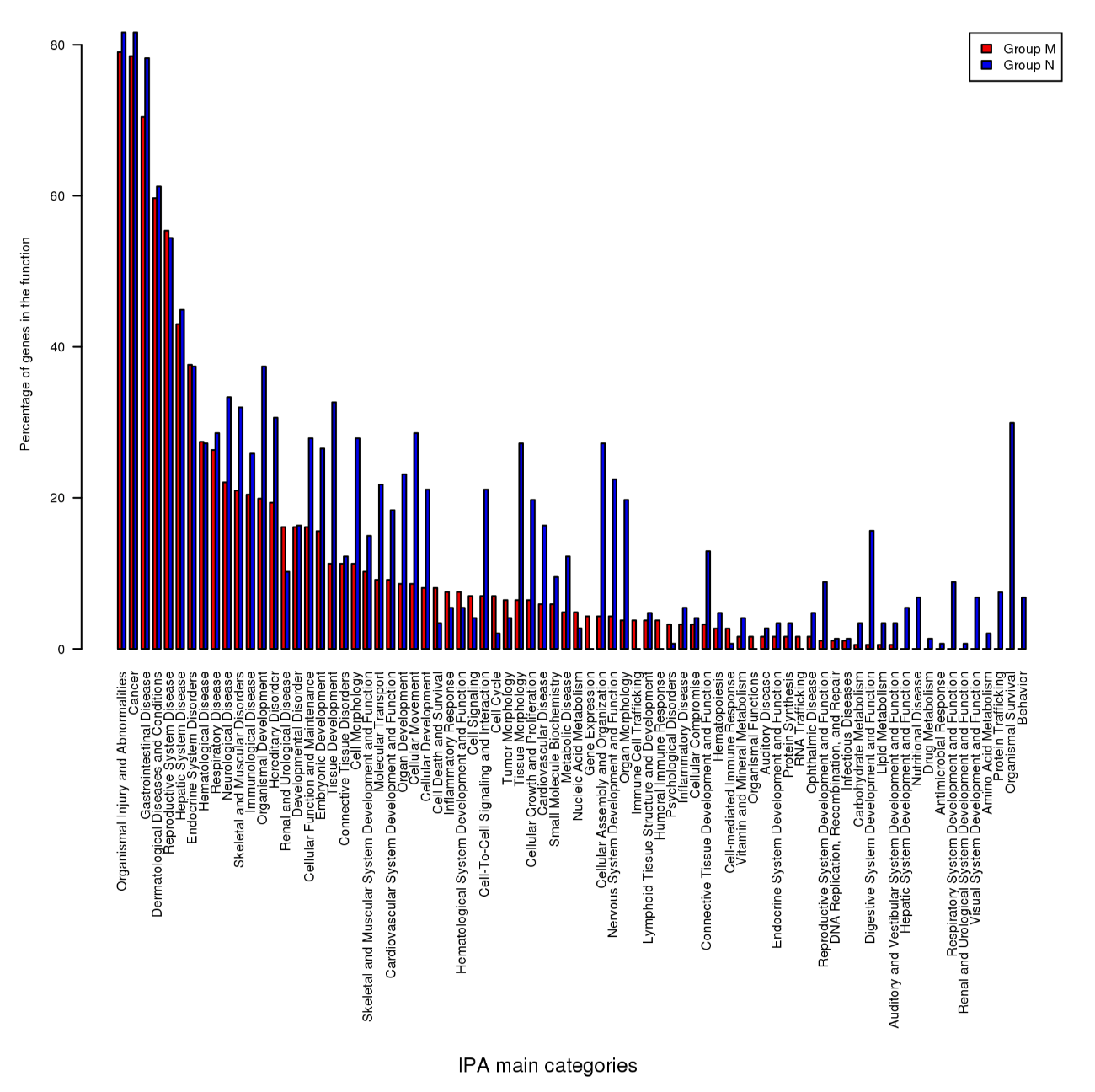

Supplement: Supplementary file 1 — Figure S1. Complete list of 56 specific pathways from PANTHER public database (identified by PANTHER specific codes) in which 139 genes (to which differentially present exons belong both from groups M and N) are classified. Figure S2. Complete list of main function categories from IPA.Table S1. cfDNA isolation from plasma and NGS. Table S2. Complete list of over‐represented genes for groups N (non‐metastatic) and M (metastatic). Table S3. Verification test. Table S4. Classification of unclassifiable (U) patients by the algorithm. Table S5. Complete list of main IPA function categories affected by over‐represented genes in group M with their associated range of P‐values. Table S6. Complete list of main IPA function categories affected by over‐represented genes in group N with their associated range of P‐values. Table S7. Complete list of IPA networks in which over‐represented genes in group M are involved with their associated scores (based on the number of over‐represented genes in the network with respect to the global size of that network). Table S8. Complete list of IPA networks in which over‐represented genes in group N are involved with their associated scores (based on the number of over‐represented genes in the network with respect to the global size of that network). [file CAM4-7-1706-s001.docx]
